# Cascading Beta-oxidation Intermediates for the Polyhydroxyalkanoate Copolymer Biosynthesis by Metabolic Flux using Co-substrates and Inhibitors

**DOI:** 10.1080/15685551.2023.2179763

**Published:** 2023-02-23

**Authors:** Geethu Madhusoodhanan, Shruthi KS, Raghu Chandrashekar Hariharapura, Divyashree M Somashekara

**Affiliations:** a Department of Biotechnology, Manipal Institute of Technology, Manipal Academy of Higher Education, Manipal, India; b Department of Pharmaceutical Biotechnology, Manipal College of Pharmaceutical Sciences, Manipal Academy of Higher Education, Manipal, India

**Keywords:** *Bacillus endophyticus*, fatty acids, inhibitors, polyhydroxyalkanoate, shake flask cultivation

## Abstract

Polyhydroxyalkanoates (PHAs) are biopolymers that are produced within the microbial cells in the presence of excess carbon and nutrient limitation. Different strategies have been studied to increase the quality and quantity of this biopolymer which in turn can be utilized as biodegradable polymers replacing conventional petrochemical plastics. In the present study, *Bacillus endophyticus,* a gram-positive PHA-producing bacterium, was cultivated in the presence of fatty acids along with beta-oxidation inhibitor acrylic acid. A novel approach for incorporating different hydroxyacyl groups provided using fatty acids as co-substrate and beta-oxidation inhibitors to direct the intermediates to co-polymer synthesis was experimented. It was observed that higher fatty acids and inhibitors had a greater influence on PHA production. The addition of acrylic acid along with propionic acid had a positive impact, giving 56.49% of PHA along with sucrose which was 1.2-fold more than the control devoid of fatty acids and inhibitors. Along with the copolymer production, the possible PHA pathway functional leading to the copolymer biosynthesis was hypothetically interpreted in this study. The obtained PHA was analyzed by FTIR and ^1^H NMR to confirm the copolymer production, which indicated the presence of poly3hydroxybutyrate-co-hydroxyvalerate (PHB-co-PHV), poly3hydroxybutyrate-co-hydroxyhexanoate (PHB-co-PHx).

## 1. Introduction

Synthetic polymers have been an integral part of our life, due to its versatility and application in all streams to improve the quality of life. The environmental hazards put forward by these synthetic polymers and their contribution to air pollution and waste management were found to be a major concern in the past two decades. The utilization of biodegradable polymer as a biomaterial for the new era gained attention in the recent years to reduce the environmental hazards. Worldwide interest to use bio-based polymers has accelerated as an eco-friendly alternative, which resembles plastic in their physio-chemical properties in order to overcome the increased demands in the rapidly developing fields like biomedical and industrial sectors [1–4]. PHAs are storage compounds produced in excess within the cytoplasm in amorphous state when carbon sources are in plenty. The carbon sources are converted into PHA and stored as granules in the cell cytoplasm. PHAs are high molecular weight, water-insoluble granules produced by assimilating carbon sources, which gets converted to hydroxyalkanoate (HA) compounds and finally polymerized within the cytoplasm [5].

It was reported that 30% of the production cost rely on the carbon source attributed to synthesize higher cases of biopolymers [2]. It is essential to manifest different sources that aid in adding copolymer that reduce the stiffness and brittleness of the biopolymer. To improve the physiochemical properties of PHA, incorporation of different hydroxy acyl unit into polymer sequence is reported to be an effective method. Random copolymers with C3 to C12 units are being synthesized by different bacterial genus depending on the PHA biosynthetic pathways, substrate provided and the functional genes residing in PHA producers. To improve the quality of PHA synthesized many attempts were experimented, out of which addition of fatty acids and plant oils were considered to be a good inexpensive carbon source [6,7]. The use of fatty acid as an additional supplement has been reported to increase the incorporation of different monomers that increase the quality of PHA [8]. The production of copolymer with a variety of functions in a large scale needs to be optimized as an essential study to use PHA as an alternative to commercial plastics and to use it for many practical uses [9]. The reduction in the biomass yield as well as low PHA content within the dry cell weight due to the incorporation of fatty acid in the medium is currently a growing concern [10,11]. Focus on PHA producers that can grow and produce PHA maximum utilizing co-substrate as oil or fatty acids can be screened to overcome the increased PHA production cost, which in turn improves the material quality of the synthesized biopolymer.

A major challenge in the biosynthesis of PHA from fatty acid (FA) is that it is an intensive process involving several iterative cycles and enzymes that are interconnected with the regular metabolism [12]. As an attempt to accumulate the major intermediates of mcl-PHAs, the modulation of fatty acid oxidation cycle can be used so as to engineer specific pathway resulting in producing quality PHA. Furthermore, the process of PHA biosynthesis from fats can be made more efficient using synthase enzyme with broad specificity [13]. In this investigation, the production, optimization and analysis of the copolymer produced by *Bacillus endophyticus* was studied in a shake flask using propionic acid (PPA) and acrylic acid (AA) and also in combination of both. The novelty of the work is that, it is the first attempt to introduce fatty acids along with inhibitors of beta-oxidation pathway in the presence of direct sucrose to elicit the copolymer production in *Bacillus* strains. The possible metabolic pathway route has also been interpreted to understand the possible PHA production within the strain.

## 2. Materials and Methods

### 2.1 PHA production combining fatty acids and inhibitors

PHA production studies were performed in the Erlenmeyer flask with 50 mL of optimized production medium (PM) (g/L): Na_2_HPO_4_ 2H_2_O- 2.2, KH_2_PO_4_ − 1.5, (NH4)_2_SO_4_ − 1.5, MgSO_4_ 7 H_2_O- 0.2 and Sucrose 20 g/L used as basic medium for co-substrates and inhibitors study. The pH of the medium was adjusted to 7 and 10% inoculum was added in all the cases. To investigate copolymer production by *Bacillus endophyticus*, fatty acids: PPA; PLA and CPA were used as co-substrates and were added at 15 mM concentration to the production medium at 36 h and 15 mM of beta-oxidation inhibitor acrylic acid (AA) was added at 48 h of cultivation. The fatty acid and inhibitor concentrations were taken based on the statistical optimization studies performed for fatty acids and AA earlier in the laboratory [14]. The combination that gave maximum PHA production was used in this study to identify the co-polymer production. The present study was performed in three batches. First, PHA production in the presence of co-substrate i.e., 15 mM of selected fatty acids (added at 36 h), second batch with inhibitor i.e., 15 mM AA (added at 48 h) and third batch with combination of fatty acids (15 mM) and AA (15 mM). All the flasks were incubated at 32°C for 72 h and the experiments were performed in duplicates. Flask without fatty acids and inhibitors are maintained as controls. After the incubation period, biomass and PHA were estimated gravimetrically [14].

### 2.2 Estimation of cell dry mass and residual sugar

The collected culture broth was centrifuged at 10,000 rpm (Plastocraft SSR-V/FM) for 10 min and the supernatant was analyzed for residual sugar by dinitrosalicylic acid method [15] through spectrophotometric analysis at 540 nm. The harvested cell pellet was washed with distilled water and then dried at 80°C to a constant weight [16].

### 2.3 Extraction of PHA

The PHA content within the cytoplasm was estimated by hydrolyzing the biomass by adding 4% of sodium hypochlorite solution [17]. The obtained hydrolysate was centrifuged and followed by water and acetone wash. Lately, the contents left after the wash were dissolved in chloroform, air dried and quantified gravimetrically [18].

### 2.4 Analysis of PHA film

For FTIR analysis, pelletization of the obtained PHA film was done by mixing up with potassium bromide (KBr). The pellet in the sample chamber of FTIR spectrophotometer was exposed to infra-red radiation with a spectral range of 4000–400 cm^−1^ (Shimadzu 8400 S). For NMR spectrum, 5 mg of PHA film was dissolved in deuterated chloroform with ^1^H NMR spectrum (Bruker Ascend 400 NMR spectrometer). DSC analysis was performed using DSC-60 plus (Shimadzu, Japan); the nitrogen flow of the instrument was 50 ml/min, which helped in minimizing the oxidative degradation of polyhydroxybutyrate (PHB) standard. A total of 5 mg of PHB standard in powder form wrapped in aluminum foil helped in minimizing thermal conductivity.

## 3. Results and Discussion

### 3.1 Fatty acids and acrylic acid co-feeding strategy to elicit co-polymer production

The capability of *Bacillus* species to produce copolymer of PHA using different carbon sources along with volatile fatty acids has been reported by different research groups [**19**,^20^]. The impact of fatty acid incorporation to the optimized production medium to evaluate growth and PHA production by *Bacillus endophyticus* was performed in the shake flask at 200 rpm for 72 h at 32°C. Based on the previously observed result, addition of co-substrate during the initial stages of stationary phase i.e., 36 h was found to have an impact on the overall PHA production compared to the addition at 48 h optimized statistically by Taguchi design [14]. In this study, the focus was given to analyse the production of PHA copolymer and to predict the possible functional pathway. Saturated fatty acids like PPA, PLA and CPA selected based on the statistical optimisation performed previously in three saturated fatty acids were added to PHA production medium separately at 36 h of cultivation; at the point where the main carbon source sucrose was expected to deplete, the organism will enter the stationery phase and where PHA biosynthetic pathway gets activated. The depletion of sucrose forces the organism to take up co-substrates i.e., fatty acid added at a later stage (after 36 h of incubation). Another set of shake flask experiment performed by adding 15 mM of AA at 48 h to PM to study the role of addition of inhibitor showed positive response towards increased production of PHA. A total of 10 ml of the production medium was extracted timely to analyse the production rate and carbon source depletion. The biomass, PHA, residual sugar and PHA % of each batch of shake flask experiment are represented in Table 1. In the last batch of the shake flask, supplementation of co-substrates along with inhibitors were studied, which enabled to understand the co-metabolism by the strain.

**Table 1.** Growth and PHA accumulation with respect to residual sugar concentration observed after adding three different fatty acids and acrylic acid at 36 and 48 h interval by *B. endophyticus.*

Sl.No	Samples	Biomass g/L	PHA g/L	PHA %	Residual sugar % after 72 h growth
1	Control 1 (2 %)	0.996 ± 0.007	0.272 ± 0.057	27.31	0.29
2	Control 2 (4 %)	1.496 ± 0.01	0.562 ± 0.095	37.57	0.227 ± 0.148
3	PPA +S (2%)	1.19 ± 0.031	0.46 ± 0.007	38.65	0.25 ± 0.017
4	PLA +S (2%)	0.867 ± 0.057	0.314 ± 0.085	36.24	0.28 ± 0.057
5	CPA +S (2%)	0.806 ± 0.095	0.267 ± 0.06	33.13	0.288 ± 0.06
6	AA+S (2%)	0.698 ± 0.09	0.259 ± 0.055	37.10	0.16 ± 0.055
7	PPA +AA+S (2%)	1.294 ± 0.09	0.731 ± 0.055	56.49	0.277 ± 0.055
8	PLA +AA+S (2%)	1.05 ± 0.09	0.436 ± 0.042	41.52	0.196 ± 0.007
9	CPA +AA+S (2%)	1.161 ± 0.042	0.529 ± 0.02	45.56	0.199 ± 0.06

PPA = Propionic acid, PLA = palmitic acid, CPA = caprylic acid, AA = acrylic acid, S = sSucrose

**Figure 1.**
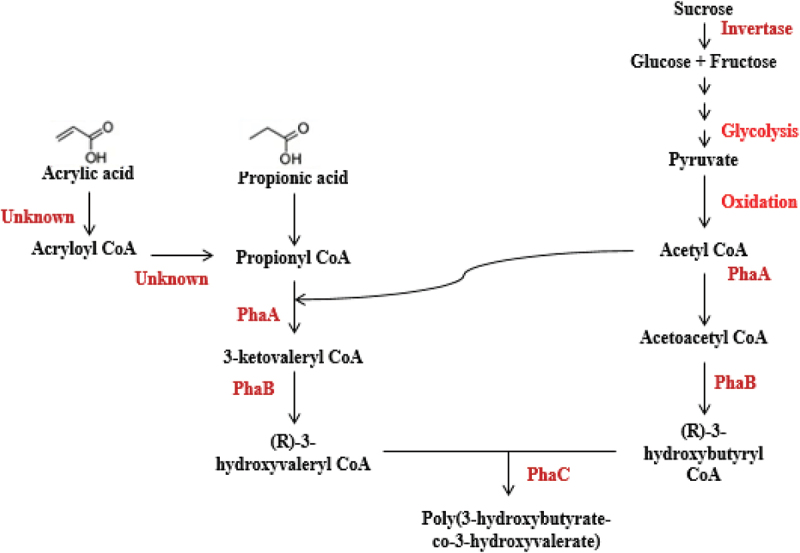
Schematic of the metabolic pathway in which PHA biosynthesis happen in the presence of PPA and AA supplementation as the co-substrate.

**Figure 2.**
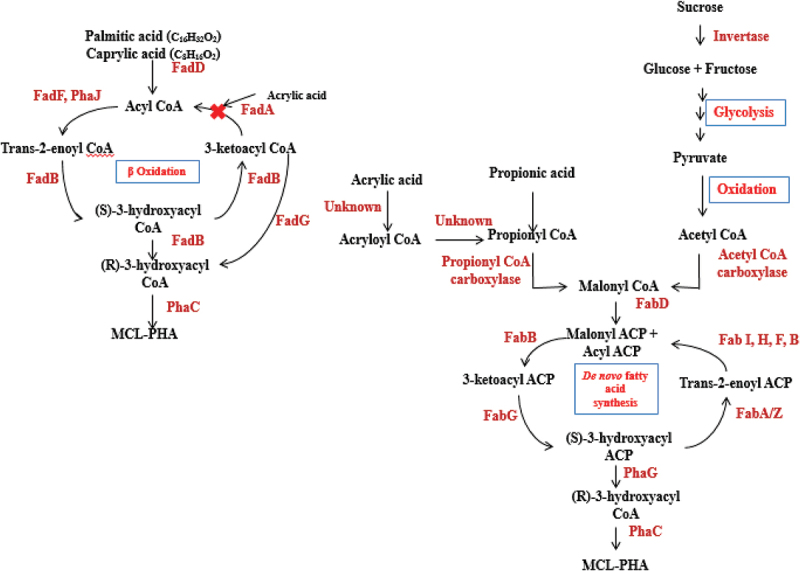
The proposed metabolic pathway schematic showing the role of fatty acid metabolism in the production of MCL-PHA using PPA, PLA and CPA as co-substrates and AA as the inhibitor.

The monomeric composition of PHA, completely rely on the substrate provided for PHA biosynthesis. From the observed result, *B. endophyticus* gave maximum production of PHA utilising saturated fatty acid especially PPA. Comparing to the control, PM medium with only sucrose could give 37% of PHA whereas in the presence of 2% sucrose and 15 mM PPA organism could produce more PHA. Similarly, PLA and CPA addition also gave increased PHA production compared to control. Cell growth remained unaffected by adding 15 mM of AA, which gave an indication that this concentration of acid was not lethal towards *Bacillus* and in turn gave PHA production more or less similar to control medium (2% sucrose) devoid of inhibitor. Compared to the PHA accumulation studies performed (Table 1), the yield after adding PPA and AA at 36 and 48 h, respectively, in an independent study PHA yield was 38.65% and 37.10%, respectively. The combination of inhibitor and fatty acid and the coexistence of fatty acid (PPA-15 mM) and inhibitor (AA 15 mM) gave increased PHA percentage of 56.49 with 1.294 ± 0.09 g/L biomass and 0.731 ± 0.055 g/L PHA (Table 1). Among all the other combinations, this combination gave maximum biomass and PHA with PPA as the co-substrate. In the presence of PPA as co-substrate in the basal medium, organisms tend to generate propionyl CoA by combining with acetyl CoA. This newly formed CoA moiety condenses to form 3-ketovaleryl CoA, which eventually gets converted to 3-hydroxy valeryl CoA. *Bacillus endophyticus* possessing PHA synthase type IV functional enzyme polymerizes monomers into polyhydroxyvalerate (P-3HV) [21]. PPA is a short-chain fatty acid, and the pathway by which it produces PHA is shown in Figure 2. Simple volatile fatty acids with less than four carbons do not enter beta-oxidation for their degradation in this case. The addition of AA may be hypothesized to form propionyl CoA as it is similar to PPA except for a double bond at the second carbon giving the highest yield. This co-feeding strategy enabled the organism in the linear consumption of both as secondary carbon source effectively to enhance PHA production relatively. The schematic of the metabolic pathway in which PHA biosynthesis occurs in the presence of PPA and AA supplementation as co-substrate is shown in Figure 1. In *Rhodobacter sphaeroides*, an enzyme Acrylyl-Coenzyme A Reductase (*acuI*) converted acrylate to propionyl CoA [22]. Therefore, AA might serve roles other than an inhibitor of 3-ketoacyl-CoA thiolase in the beta-oxidation, which needs to be tested.

The organism was capable of utilizing carbon sources provided in the form of saturated fatty acids rather than unsaturated fatty acids. Unsaturated fatty acids decrease the cell mass and PHA production compared to saturated fatty acids. These findings were reported by Lo et al., in 2005, in which *S. natans* showed increased cell growth in the presence of saturated fatty acids like palmitic and steric acids [23]. The double bond in the unsaturated fatty acids make these co-substrates less available to the organism due to reduced 2,4-dienoyl CoA reductase activity, which was mentioned by Srivastava and Tripathi in 2013 [24]. The cell mass density of the organism remained unaffected, whereas the PHA conversion rate was less compared to the supplementation of unsaturated fatty acids and control. The higher carbon fatty acids like palmitic and caprylic acids gave PHA, indicating that the organism can breakdown and utilize these sources to synthesize PHA. This studies clearly interpret that the depletion of direct carbon source (sucrose) elicit the organism to uptake fatty acid provided via carbon source, which in turn diverts organisms to synthesize PHA with more copolymers in the monomeric units. The efficiency of *B. endophyticus* in taking up higher fatty acids needs to be analyzed thoroughly to study the metabolic pathway and the functional enzymes active in producing the copolymer. The material properties of the PHA film obtained during the study was analyzed by FTIR and ^1^H NMR to understand the copolymer production brought about by the fatty acid addition [25].

PPA was reported to be a good precursor to initiate PHA copolymer production with the optimal production rate and also in incorporating 3-hydroxy valerate (3HV) content in the biopolymer synthesized [19,21,26]. Many research groups focused on different feasible strategies to alter the biomaterial property by varying the concentration of the co-substrate. Attempts were carried out to combine the PHA biosynthetic gene and cell growth genes to increase the monomer composition along with the addition of higher fatty acids. The copolymer of 3-hydroxybutyrate (3HB) and 3HV was extensively studied and produced by adding PPA and pentanoic acid by *Ralstonia eutropha*. The incorporation of the valerate unit made the synthesized biopolymer more flexible and was reported to improve the material properties [27,28]. Lactic and acetic acids were reported to be an effective carbon source to accumulate PHA as most of the PHA producers can assimilate these acids in a higher rate [8,29,30]. In our study, saturated fatty acids like PPA addition to the medium gave maximum co-polymer as well as PHA production. The efficiency of *B. endophyticus* to take up higher fatty acid was analyzed, which helped in understanding the metabolic pathway and the functional enzymes active in producing the copolymer. The role of higher fatty acid and the metabolic pathway that leads to the production of medium chain length (mcl) PHA using PPA, PLA and CPA as co-substrates are depicted in Figure 2.

Production of PHA by *Bacillus* strain is due to the polymerization process mediated by the PHA synthase enzyme coded by the *PhaC* gene using hydroxyacyl CoA monomers releasing CoA units. The monomeric subunit for the process is usually generated as the end result of two pathways [31]. The most common is the dimerization pathway involving two acetyl CoA moieties involving *PhaA* and *PhaB* gene coding 3-ketothiolase and acetoacetyl CoA reductase, respectively. Fatty acid beta-oxidation pathway is another alternative cycle that releases monomeric units for PHA biosynthesis. Chemical inhibition of the enzymes has also been reported to be successful in accelerating the biosynthesis of PHA from fatty acids. Retarding beta-oxidation pathway by deleting *fadA* or *fadB* enzyme along with chemical inhibition has been a widely used alteration of PHA biosynthesis involving fatty acids [32]. The beta-oxidation pathway has been modified to increase PHA biosynthesis in two ways, one method is to inhibit the key enzymes involved in the beta-oxidation pathway enzymes such as *fadA* and *fadB* and the second method involves the amplification of enzymes like reductase and hydratase that are diverting the conversion of beta-oxidation pathway intermediate to its corresponding R3HA CoA [33]. Usually beta-oxidation-mediated pathway that is reported to enhance the release of monomeric units occurred only in those organisms that possess hydratase enzyme coded by *PhaJ*. This enzyme is involved in converting trans-2-enoyl CoA directly to R3HA CoA. The presence of *PhaJ* or *PhaJ* mediated PHA biosynthesis is mainly responsible for the mcl PHA production. Till date, short and mcl enoyl CoA specific types of *PhaJs* were reported [34].

Recently, as the result of whole-genome sequencing, few *Bacillus* strains were reported to possess the *PhaJ* gene in the PHA gene cluster. *Bacilus cereus, Bacillus thuringiensis* and *Bacillus anthracis* were reported to possess the *PhaJ* gene in the *Pha* gene cluster and this gave new insights towards the capability of *Bacillus* strain to utilize higher fatty acids to produce mcl polymers mediated by beta-oxidation-generated monomeric units. In most of the *Bacillus* strains, this gene is present to code for Mao-C like proteins and the same gene when present in PHA-producing *Bacillus* strains possibly owns the hydratase activity. The capability of *Bacillus* species to produce the copolymer of PHA using different carbon sources along with volatile fatty acids has been reported by many research groups [20,35]. It is essential to determine the quality of biopolymer produced by *B. endophyticus* that can ensure to meet the standards required to replace synthetic plastics. So in the present study, providing higher fatty acids as co-substrates and inhibitors possibly blocks the regular beta-oxidation pathway enzymes and elicits the hydratase activity of *PhaJ* gene so as to accumulate favorable monomers that tend to increase the quality of the polymer, which is represented in Figure 2 showing that the possible route of higher fatty acids and inhibitors act on accumulating the desired monomers for mcl PHA biosynthesis in the current experiment. To modulate the metabolic flux for the novel copolymer production in *B. endophyticus*, the addition of different fatty acids as co-substrate in the production medium was found to have an influence on PHA production.

The role of inhibitors in PHA metabolism is to channelize the intermediates towards specific pathways by blocking enzymes functional in the fatty acid beta-oxidation so as to enhance PHA biosynthesis. Combining fatty acids that gave maximum PHA production along with fatty acid inhibitors like AA was performed in the shake flask to study the interactive effect of incorporating higher carbon chain in the polymer structure. AA, fatty acid beta-oxidation inhibitor was noted to influence PHA production positively in the previous phase of our study leading to increase in biopolymer production. Inhibitors like AA were reported to play a crucial role in diverting enzymes in central metabolic pathway and accumulating precursors especially in beta-oxidation pathway can be further converted to 3HA CoA, which is an immediate precursor for PHA biosynthesis [11,22,36,37].

Fatty acid accumulation brought about by the addition of inhibitors in the cell results in the production of PHA in superior amounts was observed in this study. Usually, inhibitors above a particular amount can be inhibitory towards bacterial growth. So the simultaneous addition of inhibitor and fatty acid at 48 and 36 h of growth phase can accelerate PHA production by diverting the usual pathway of the organism to PHA pathway. The results for this experiment are represented in Table 1. It was found that few inhibitors and fatty acids were taken up by the test organism for PHA biosynthesis at 48 h, concluded from our previous studies. Mostly, the PHA produced with the incorporation of sucrose is mostly a homopolymer of P-3-hydroxybutyrate (P3HB). Due to high crystallinity (55–80%), P3HB is highly crystalline and brittle, limiting its applications. So the incorporation of monomer types helps in reducing the shortcomings of the biopolymer. There are reports that coplymer P(3HB-co-HV) exhibit lower crystallinity and reduced stiffness, which makes it available for wider appliations [26].

### 3.4 FTIR

**Figure 3.**
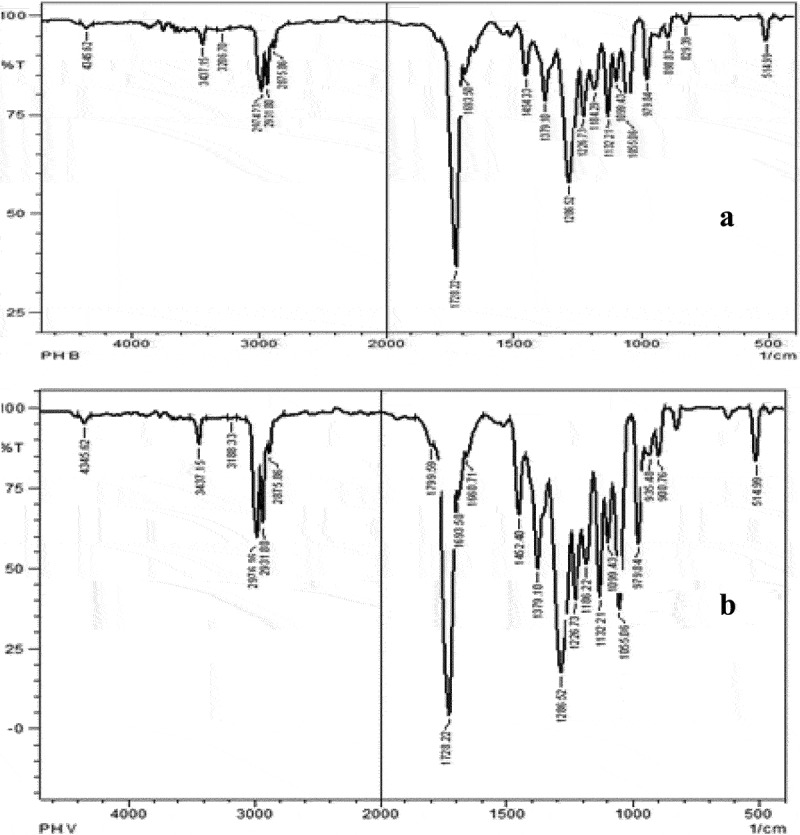
FTIR spectra of standard (a) PHB and (b) P (HB-co-HV).

Fourier transform infrared spectroscopy has been demonstrated to be a useful technique to detect PHA and to study the cell components in the intact form. FTIR spectra reflect the proportions of functional groups present in the sample and is typically used as a tool to provide information on the polymer structure. The degree of crystallinity of the polymer was effectively analyzed by studying FTIR spectrum by Valappil *et al*., in 2007, which ended up in characterizing the PHB produced by newly isolated *Bacillus* strain, *Bacillus cereus* SPV [38]. The absorption bands (Figure 3) observed at 1728 cm^−1^ corresponding to ester carbonyl group and the characteristic – CH group peak observed at 1282 cm^−1^ characteristic of PHB [39] were identified in the polymer.

**Figure 4.**
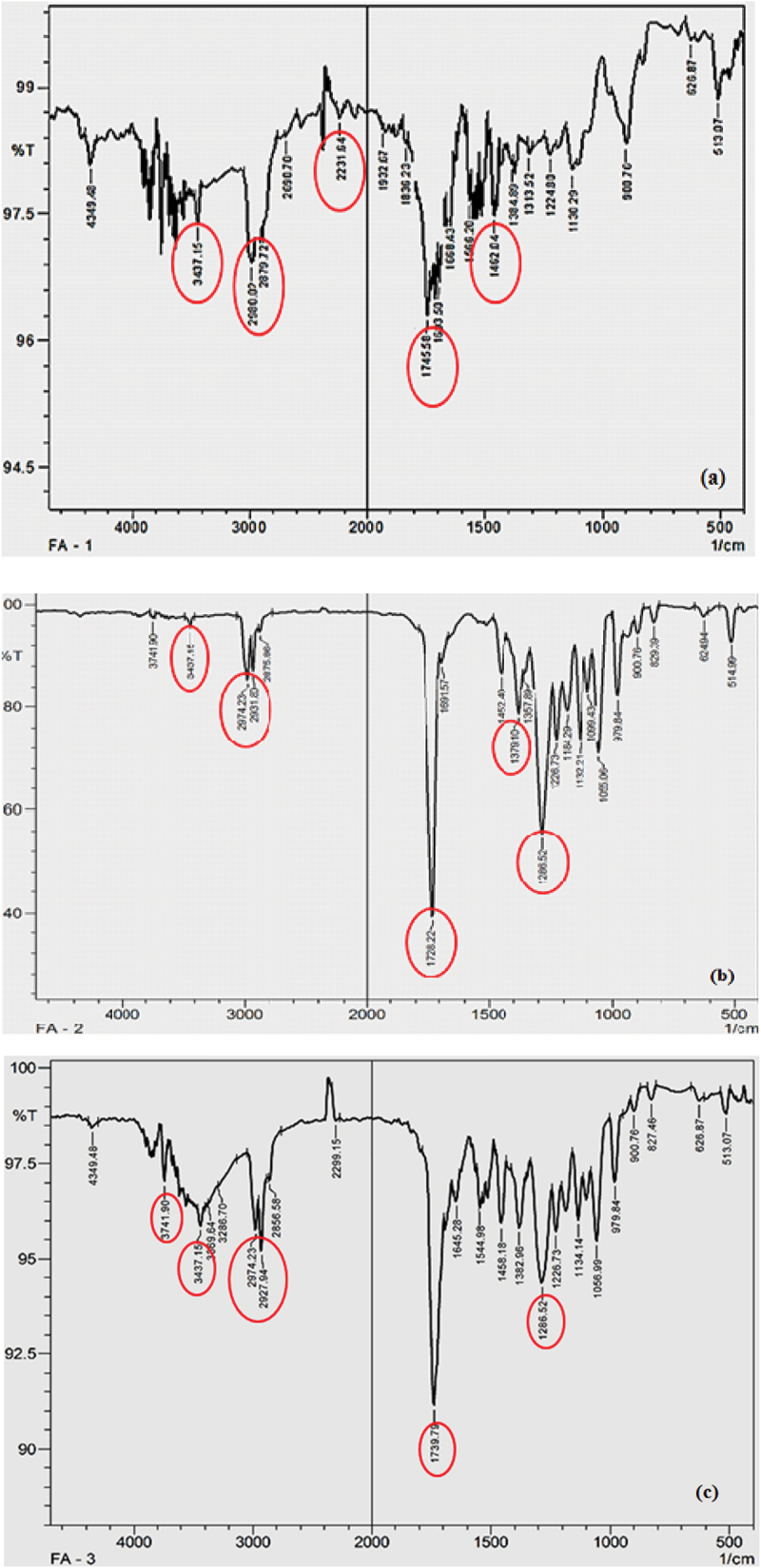
FTIR spectra of PHA film isolated from *Bacillus endophyticus* using substrate 2% sucrose, co-substrate (a) PLA, (b) CPA, (c) PPA along with AA as inhibitor (15 mM) added at 48 h of cultivation.

The PHA film obtained after the experimental trial had characteristic peak at 2953 cm^−1^, 2766 cm^−1^, 2349 cm^−1^, 1711 cm^−1^, 1531 cm^−1^, 1046 cm^−1^, 500–1000 cm^−1^ and 1679 cm^−1^ corresponding to C–H methylene bunch, C–H extend, –C:C–stretch, C=O extend, –C = C–stretch, N–O unbalanced extend, C–O extend and OH, respectively. An exceptionally highest peak crest at 1711 cm^−1^ was seen in few PHA samples corresponding to ester carbonyl (C = O) bonds, which was identified as an extended vibration of PHB. These FTIR characteristic peak crests were precisely seen in those PHA film obtained by co-feeding the strains with FA and inhibitor. The results are shown in Figure 4 (a, b and c), in which the prominent peaks were highlighted which confirmed PHA produced by comparing with the standard PHB and PHB-co-PHV peaks shown in Figure 3 (A and B). Characteristic mcl peak i.e., C-H methylene vibration seen at 2927 cm^−1^ was with the PHA film produced by cultivating along with PPA. This strong vibration around 2933–2972 cm^−1^ indicates the presence of the PHB-co-PHV co-polymer. The PHA film obtained after the experimental trial gave intense stretching vibration around 1724 cm^−1^ representing C = O group confirming PHA.

### 3.5 5 ^1^H NMR

**Figure 5.**
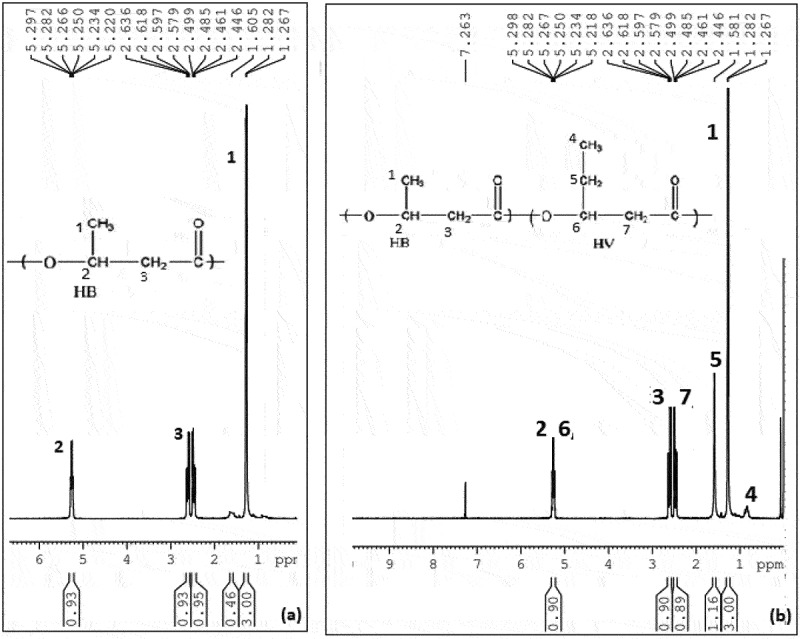
^1^HNMR spectra of standard (a) PHB and (b) P (HB-co-HV).

**Figure 6.**
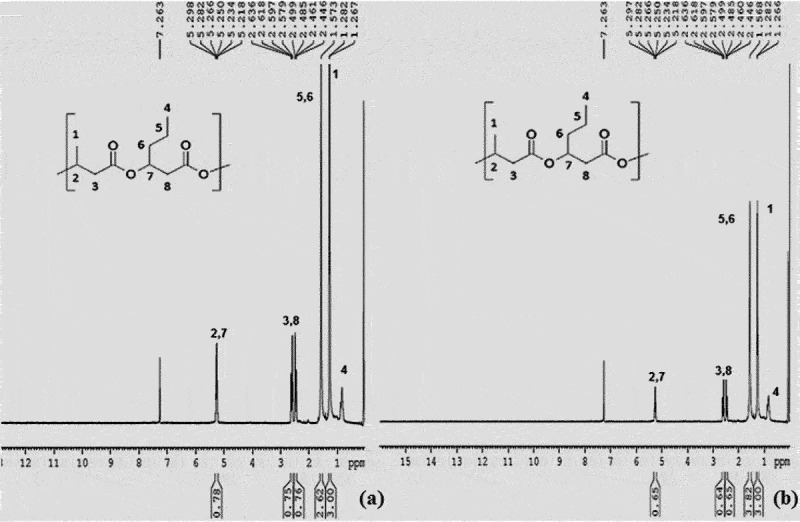
^1^H NMR spectra of PHA film isolated from *Bacillus endophyticus* using 2% of sucrose (substrate), co-substrate as (a) CPA (15 mM), (b) PPA (15 mM) and AA as inhibitor (15 mM) added at 48 h of cultivation.

**Figure 7.**
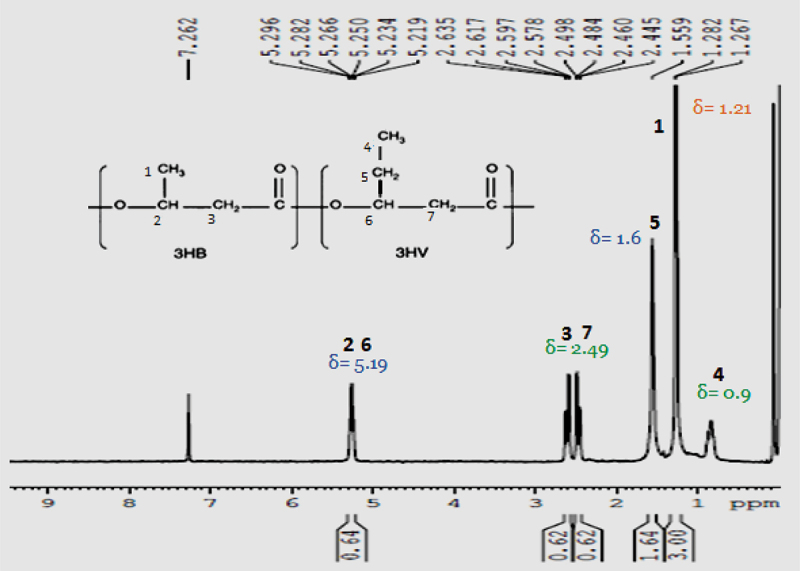
^1^H NMR spectra of PHA (45.5% of PHA) extracted from *Bacillus endophyticus* using substrate 2% sucrose and co-substrate as PPA (15 mM).

^1^H NMR was a characteristic analysis done for all PHA films produced by *B. endophyticus* using sucrose and fatty acids as co-substrates and the analysed ^1^H NMR spectrums usually show three characteristic groups signals of PHB: A doublet at 1.29 ppm, a doublet of a quadruplet at 2.5 and a multiplet at 5.28 ppm corresponding to methyl, methylene and methyne groups, respectively. The ^1^H NMR spectrum of PHA film showed all these three characteristic group signals of PHB with a doublet at 1.29 ppm and a doublet of a quadruplet at 2.5 ppm. The quadruplet attributed towards methylene group, which was near to an asymmetric carbon bearing single atom. All NMR spectra were compared and confirmed with the standard PHB and PHB-co-PHV represented in Figure 5 (a and b), respectively. The entire spectrum showed similar spectral lines as that of PHB standard attributing to methyl and methylene groups coupled with single proton. A characteristic triplet at 0.9 ppm, resonance at 1.59 and at 5.15 indicated the presence of valerate and represented in Figure 7. ^1^HNMR spectra of the PHA film obtained after adding PPA as co-substrate confirms that our test strain is capable of synthesizing PHB and PHV. The H^1^ NMR spectra of PHA obtained after adding PLA and CPA is represented in Figure 6, which showed spectra corresponding to Poly3hydroxy butyrate-co-hydroxyhexanoate synthesis from PHA synthesized using fatty acids palmitic and caprylic acid along with AA. The molar percentage of hydroxy butyryl unit in the copolymer was 96.4% and hydroxyvalerate was 3.6%, which confirms the polymer as PHB-co-PHV in the presence of fatty acid even with the 15–20 mM range (Figure 7). The range was also metioned by Kulkarni et al., in 2010 in PHA produced by *Halomonas campisalis MCM B-1027* strain [40].

### 3.6 DSC analysis

Differential scanning calorimetry analysis was performed for the PHB standard. It was used to measure the melting temperature (T*_m_*) of the standard polyhydroxybutyrate. The DSC analysis of our samples was performed using the DSC-60 plus (Shimadzu, Japan) instrument. The temperature was ramped at 350°C under nitrogen. The standard used obtaining the DSC peak was polyhydroxybutyrate and PHB-co-PHV, and this can be used to compare the biopolymer synthesized by *B. endophyticus* later by maintaining the same program [41].

**Figure 8.**
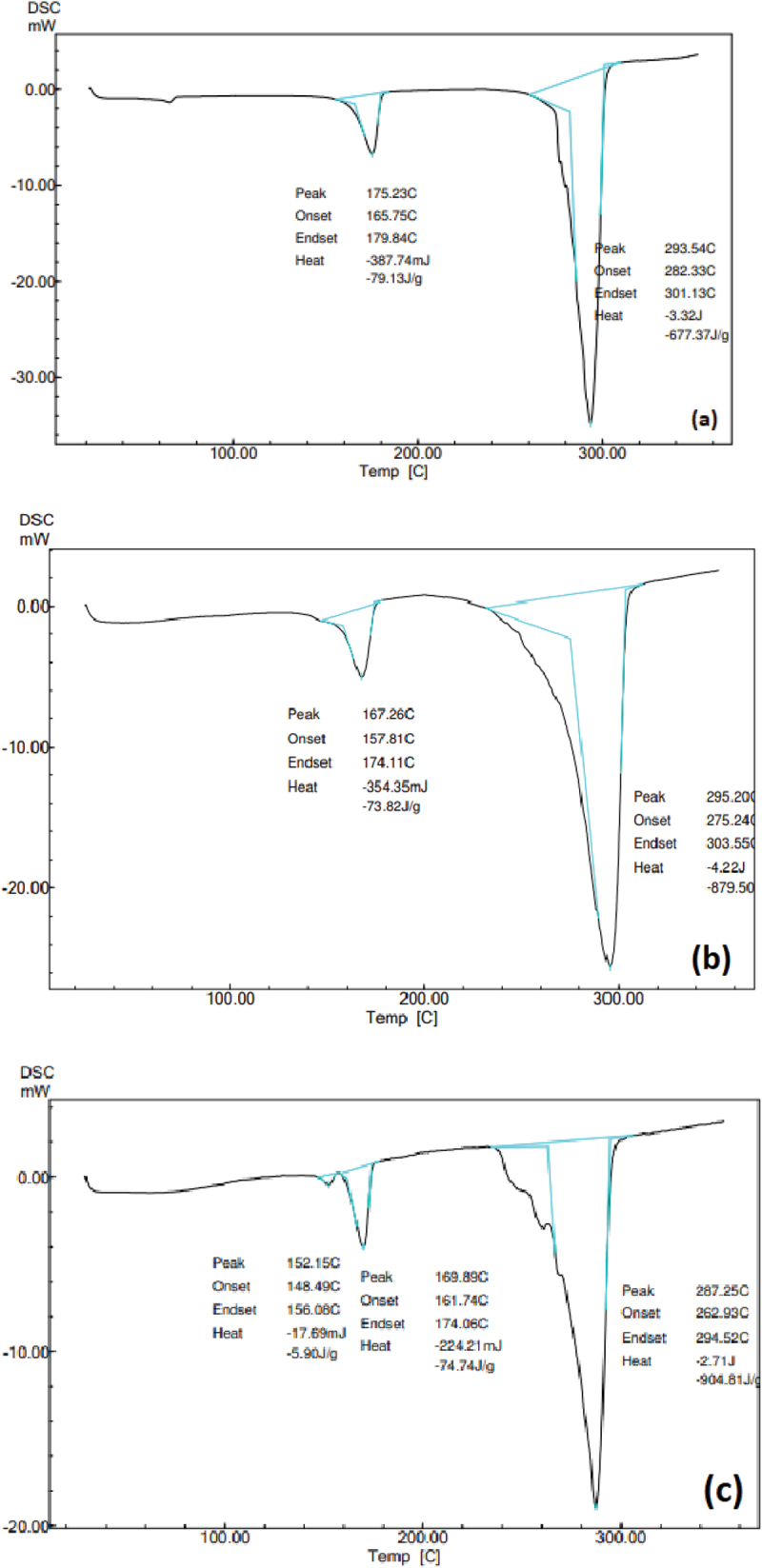
DSC thermo grams of (a) standard PHB, (b) PHA obtained from CPA as substrate and (c) PHA obtained from PPA.

DSC analysis was performed and the nitrogen flow of the instrument was 50 ml/min, which helped in minimizing the oxidative degradation of PHB standard. A total of 5 mg of PHB standard in the powder form were wrapped in aluminum foil helped in minimizing thermal conductivity. The DSC output gave melting temperature details of the polymer. From literature, the melting temperature (T*_m_*) value of the standard showed a characteristic peak value at 175°C [41]. The PHB standard also gave a peak value at 175.23°C (Figure 8a). T*_m_* is an important characteristic of polymer. Crystallization and melting are two different transitions with first order and show as a peak in DSC curve. Crystallization of the polymer usually shows as positive peak, which is an exothermic reaction. Followed by crystallization is the melting temperature of polymer usually shown as negative peak in the curve.

PHA copolymers were reported to have different physical and thermal properties. The thermal properties like melting temperature (T*_m_*) and thermal degradation temperature (T*_d_*) determine the temperature conditions on which it can be processed. The physical and chemical properties of PHA are largely depended on the mol % of the copolymers.

It was reported that the T*_m_* of the ideal polymer should be below its degradation temperature. The melting and degradation temperature of PHA polymers produced by *B. endophyticus* in the presence of substrate (sucrose), co-substrate (FAs) and inhibitor (AA) are represented in Table 2. The melting temperature of the polymers was in the range of 150–175°C. In this study, copolymers with mcl monomers displayed a decrease in the melting temperature. In this case, PHA produced in the presence of PPA gave T_*m*_ of about 167°C (Figure 8b), whereas with HV monomer displayed a lower T*_m_* than the standard. According to the literature, when the monomers vary and the mol % of PHB content in the copolymer differs, it always resulted in variation in the T*_m_* compared to the standard. Melting temperature is more correlated with the presence of mcl monomers especially if 3HHx or 3HV content is present and T*_m_* tends to decrease. In the present study, copolymers with 3HHx content had T*_m_* of 152°C (Figure 8c) . According to Sharma et al 2017, lower T*_m_* was reported with higher mole (72) % of hexanoic acid than with PHAs produced with 42% of 3HHx [42]. The T*_g_* or degradation temperature in our study was much higher than T*_m_* in all cases and also compared to the standard PHB. The T*_d_* or degradation temperature in our study was much higher than Tm in all cases and also compared to the standard PHB (Table 2)

**Table 2.** Melting temperature and degradation temperature of PHA copolymers produced in the presence different fatty acids and inhibitors.

	Carbon sources for PHA production	Inhibitor added (15 mM)	Melting temperature (T*_m_* in ◦C)	Degradation temperature (T*_d_* in ◦C)
1	CPA (15 mM) and S (2%)	AA	167	295
2	PLA (15 mM) and S (2%)	AA	172	287
3	PPA (15 mM) and S (2%)	AA	152	252
4	S (2%)	-	175	265
5	S (4 %)	AA	174	282

PPA = Propionic acid, PLA = palmitic acid, CPA = caprylic acid, AA = acrylic acid, S = sucrose

## 4. Conclusion

Gram-positive *B. endophyticus* capable of producing PHA isolated from soil was used further to check on its capability to stimulate the production of copolymers in the presence of fatty acid, inhibitors and combination of both. The high range of copolymer production by this organism was reported to be first in the presence of higher fatty acids like CPA, PLA and PPA. Many different *Bacillus* strains were reported to produce PHA in high quantity. Comparing to those strains, our strain gave better copolymer production even in the presence of 15 mM of fatty acids and inhibitors. The inhibitor AA, well known for beta-oxidation pathway blockers, did not have a negative impact on the PHA biosynthesis, whereas enhanced the production and resulted in producing high-quality PHA. The functional PHA pathway during the biosynthesis was discussed and the capability of the organism to synthesize mcl PHA was confirmed. This strain can be used in industries to produce polymers of high quality and quantity in the presence of this optimized substrate concentration. This study serves as the promising basic to predict the possible PHA pathway functional in mcl-PHA-producing *Bacillus* strain, which is very new in this field of research. The biomedical application of higher-quality PHA especially short chain length and mcl PHAs is very relevant and useful in the current era.
